# Laparoscopic system for simultaneous high-resolution video and rapid hyperspectral imaging in the visible and near-infrared spectral range

**DOI:** 10.1117/1.JBO.25.8.086004

**Published:** 2020-08-28

**Authors:** Hannes Köhler, Axel Kulcke, Marianne Maktabi, Yusef Moulla, Boris Jansen-Winkeln, Manuel Barberio, Michele Diana, Ines Gockel, Thomas Neumuth, Claire Chalopin

**Affiliations:** a University of Leipzig, Innovation Center Computer Assisted Surgery, Leipzig, Germany; b Diaspective Vision GmbH, Am Salzhaff, Germany; c University Hospital of Leipzig, Department of Visceral, Thoracic, Transplant, and Vascular Surgery, Leipzig, Germany; d IHU-Strasbourg Institute of Image-Guided Surgery, Strasbourg, France

**Keywords:** hyperspectral imaging, laparoscopy, minimally invasive, visible and near-infrared, color video

## Abstract

**Significance:** Hyperspectral imaging (HSI) can support intraoperative perfusion assessment, the identification of tissue structures, and the detection of cancerous lesions. The practical use of HSI for minimal-invasive surgery is currently limited, for example, due to long acquisition times, missing video, or large set-ups.

**Aim:** An HSI laparoscope is described and evaluated to address the requirements for clinical use and high-resolution spectral imaging.

**Approach:** Reflectance measurements with reference objects and resected human tissue from 500 to 1000 nm are performed to show the consistency with an approved medical HSI device for open surgery. Varying object distances are investigated, and the signal-to-noise ratio (SNR) is determined for different light sources.

**Results:** The handheld design enables real-time processing and visualization of HSI data during acquisition within 4.6 s. A color video is provided simultaneously and can be augmented with spectral information from push-broom imaging. The reflectance data from the HSI system for open surgery at 50 cm and the HSI laparoscope are consistent for object distances up to 10 cm. A standard rigid laparoscope in combination with a customized LED light source resulted in a mean SNR of 30 to 43 dB (500 to 950 nm).

**Conclusions:** Compact and rapid HSI with a high spatial- and spectral-resolution is feasible in clinical practice. Our work may support future studies on minimally invasive HSI to reduce intra- and postoperative complications.

## 1 Introduction

Hyperspectral imaging (HSI) has been used in many fields of biomedical research and clinical applications in recent years.^1^ Promising results were reported for the estimation of organ perfusion,^2^^,^^3^ differentiation of anatomical structures,^4^^,^^5^ wound assessment,^6^^,^^7^ and the detection of cancerous tissue^8^^–^^11^ in animal experiments and clinical studies.^12^ However, the majority of the described systems do not apply to current minimally invasive surgery. Supporting the estimation of tissue perfusion or detection of risk structures with spectral imaging would be particularly important in laparoscopic procedures where surgeons are limited to their visual impression.

Reported multi- and hyperspectral endoscopic systems since 2013 are listed in Table 1. Two systems are capable of investigating both the visible and near-infrared range, but are limited by long acquisition times and without color video during spectral imaging or low spectral resolution.^13^^,^^14^ Simultaneous hyperspectral data and color video acquisition in the visible range were achieved with pushbroom and manual line-scanning flexible endoscopes.^15^^,^^16^ Most systems are using spectral scanning methods like acousto-optical tunable filter (AOTF), liquid crystal tunable filter (LCTF), filter wheels, or light sources with monochromators. Spectral scanning typically results in high spatial resolution but longer acquisition times per spectral channel and misalignments due to motion. Spectrally resolving detector arrays (SRDA) or snap-shot imagers are nonscanning systems and are, therefore, not limited by motion artifacts.^13^ However, spatial and spectral resolutions are reduced. Spatial or pushbroom scanning allows high spectral and spatial resolution at the same time. Previously described endoscopic pushbroom systems have the disadvantage of having to move the sample or the endoscope itself.^14^^,^^16^

**Table 1:** Specifications of reported multi- and hyperspectral laparoscopic (HSI/MSI) systems since 2013.

Author	System description [light source]	Spectral separation	Spectral range (nm)	Spectral channels [resolution in nm]	Image size in pixels [spatial resolution]	Acquisition [processing] time in s	Color video^a^	Medical application
Clancy et al.^21^	MSI laparoscope [xenon]	LCTF	500 to 620	13 [7 to 20]	512×384 [0.7 mm]	7 [45]	No	Perfusion
Fawzy et al.^22^	MSI flexible endoscope [xenon]	Filter wheel	400 to 760	18 [15]	200×200 [n.a.]	0.07 [2.4]	No	Perfusion
Hohmann et al.^23^	MSI flexible endoscope [xenon]	n.a.	400 to 650	6 [12 to 20]	350×370 [50 to 500 μm]	0.45 [1.5]	No	LG CA
Luthman et al.^13^	MSI flexible endoscope [LED]	2 × SRDA	470 to 630	16 [9 to 26]	n.a. [300 to 400 μm]	0.01 [600]	Yes	U/LG CA
			600 to 1000	25 [7 to 15]				
More et al.^24^	MSI rigid endoscope [halogen]	Line scanning monochromator	480 to 705	16 [15]	1392×1024 [n.a.]	20 [n.a.]	No	Mice retina
Zhang et al. ^25^	MSI laparoscope [n.a.]	Filter wheel	470 to 700	8 [20 to 25]	300×300 [6.45 μm]	0.4 [n.a.]	No	Tissue classification
Leitner et al.^26^	MSI + HSI rigid endoscope [xenon]	AOTF	400 to 650	MSI: 8	1004×1002 [n.a.]	MSI: 0.2	No	Classification of cancerous tissue
				HSI: 51 [5]				
						HSI: 1.25 [n.a.]		
Baltussen et al.^14^	HSI laparoscope [halogen]	2 × push-broom	400 to 1000 and 900 to 1700	n.a. [3 and 5]	1×1312 and 1×320 [n.a.]	20 and 30 [60]	No	LG CA
Han et al.^27^	HSI flexible endoscope [xenon]	Filter wheel	405 to 665	27 [10]	582×752 [n.a.]	4.2 [n.a.]	No	LG CA
Kumashiro et al.^15^	HSI flexible endoscope [xenon]	Push-broom	405 to 750	70 [5]	640×480 [n.a.]	30 [60]	Yes	LG CA
Regeling et al.^28^	HSI flexible endoscope [n.a.]	Monochromator	390 to 680	30 [10]	1388×1040 [n.a.]	6 to 9 [30]	No	HN CA
Yoon et al.^16^	Flexible HSI endoscope [LED, halogen]	Manual line scanning	450 to 710 (due to light source)	75 [9] at 18 μm slit; 50 lines/mm	n.a. [120 μm at 5 mm distance]	0.05/line [0.85/line]	Yes	UG CA
Presented system	HSI laparoscope [LED, xenon, halogen]	Push-broom	500 to 1000	100 [5]	640×480 [320 μm at 50-mm distance and 500 nm]	4.6 [4.6]	Yes	UG CA

AOTF, acousto-optical tunable filter; LCTF, liquid crystal tunable filter; SRDA, spectrally resolving detector array; U/LG CA, detection of cancers in the upper/lower gastrointestinal tract; and HN CA, head and neck cancer detection.

a Simultaneous acquisition of HSI/MSI and color video (>15  fps).

Here, the presented laparoscopic system provides a high-resolution color video and simultaneous HSI with high spatial and spectral resolution in the visible and near-infrared range. To the best of our knowledge, it is the first system with these capabilities. Furthermore, the typical limitation of pushbroom scanning is overcome for the first time in a visible and near-infrared laparoscope. Moving the spectrograph with a miniaturized stepper motor inside the housing of the laparoscopic camera allows HSI without moving the sample or the laparoscope. This paper aims to describe an HSI laparoscope with high practicability for clinical use and to evaluate it with different light sources. Additionally, HSI was performed on a human tissue resectate with the presented system and a previously described hyperspectral camera for open surgery for comparison.^17^

## 2 Methods

The HSI laparoscope was evaluated in a laboratory set-up with different light sources and variable working distances to the measured reference objects. Furthermore, data from human tissue resectates were acquired with the proposed system and a commercially available HSI system for comparison.

### 2.1 Description of the HSI Laparoscope

**Fig. 1:**
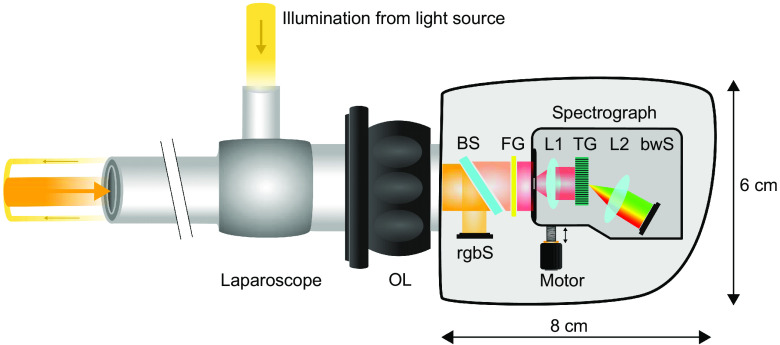
Schematic description of the laparoscopic hyperspectral camera system. The light of an external light source is coupled into the laparoscope for object illumination. An OL adapter focuses on different working distances and connects the laparoscope with the camera housing. The incident light of the visible spectral range is partially reflected from a BS on a rgbS. The remaining part of the visible light and all near-infrared light passes the BS and the high-pass FG toward the entrance slit of the spectrograph. The spectrograph consists of two achromatic lenses (L1 and L2), a transmission grating, and a monochromatic camera sensor (bwS). A stepper motor is moving the spectrograph during HSI (push-broom scanner).

The overall system is composed of an HSI and color video camera system combined in a three-dimensional (3-D) printed housing with dimensions $8\times 6\times 6\;{\mathrm{cm}}^{3}$ and a rigid laparoscope (HOPKINS^®^ 8711 AGA, KARL STORZ SE & Co. KG, Tuttlingen, Germany) with a 10-mm diameter suitable for white light, near-infrared fluorescence, and autofluorescence imaging. The laparoscope is attached to an objective lens (OL) adapter with a focal length of 14 mm and a C-mount connection on the camera side. The weights of the camera and the used laparoscope (with OL adapter) are 259 and 254 g, respectively. For illustration of the miniaturized camera system components, see Fig. 1. A beam splitter (BS) is partially reflecting the incident visible light only, while the remaining light is transmitted. The reflected light is captured by a color image sensor (IMX290LQR-C, Sony, Tokyo, Japan) with 1920×1080 maximum number of effective pixels at 120 frames per second (fps). After the transmitted light went trough a high-pass filter glass (FG) (GG 495, Schott AG, Mainz, Germany), only light above 500 nm is passing the entrance slit of the spectrograph. Two achromatic lenses are used for focusing and a transmission grating splits up the light that is captured by a monochrome camera sensor (IMX290LLR-C, Sony, Tokyo, Japan). This sensor has a relatively high quantum efficiency in the near-infrared range and can capture 120 fps with full high-definition (HD) resolution. The electronic components of the camera are controlled by a system on module (i.MX8M Quad 1.5 GHz ARM Cortex-A53, NXP Semiconductors, Eindhoven, Netherlands) and the acquired raw images are transferred via gigabit ethernet to a Windows machine (2.11 GHz Intel Core i7) for processing. During scanning, 960×780  pixels (spatial×spectral) of the maximum 2048×1088  pixels of the image sensor are read out at 140 fps. After spectral calibration of the spectrograph with a krypton lamp once, each of the 780 pixels in the spectral dimension can be assigned to one of 500 channels between 500 and 1000 nm. This 960×500 raw image is binned to 480×100 (spatial×spectral) pixels for noise reduction by averaging two pixels in the spatial dimension and five pixels in the spectral dimension. A detailed description of the used calibration and balancing process can be found by Holmer et al.^18^ The spectrograph is guided by a linear rail to ensure the axis alignment while being moved by an attached stepper motor with a diameter of 15 mm. For the acquisition of a hyperspectral data cube, the spectrograph is moved over the image plane by the stepper motor while 640 images are recorded. This results in a three-dimensional array with 640×480  pixels in the spatial dimension and 100 spectral channels with 5-nm resolution obtained in 4.6 s. Image sizes up to 1920×1080  pixels in the spatial dimension are possible, but require longer acquisition time. Data processing (from raw data to a false-color image) and scanning are performed in parallel, and therefore, no additional time is needed for computation. Furthermore, real-time visualization of the processed false-color image is available during scanning. The field of view (FOV) and the spatial resolution are dependent on the distance to the object and the focal length of the OL. The FOV was measured for 5- and 10-cm distances, and this yielded $55\times 48\;mm^{2}$ and $100\times 83\;mm^{2}$, respectively.

Even though a color image from the rgbS and a false-color image calculated from spectral data show different areas of the object, the arrangement of both systems inside the camera is fixed. This allows the registration of both images after a one-time calculation of the homography. For this purpose, 25 corresponding points are manually annotated in both images. These points are the input for the OpenCV function findHomography^19^ that uses RANSAC^20^ to calculate the homography (3×3 transformation matrix) between two planes by minimizing the back-projection error. Once the homography is estimated, the images are registered according to

$$D(x,y)=S(\frac{H_{11}x+H_{12}y+H_{13}}{H_{31}x+H_{32}y+H_{33}},\frac{H_{21}x+H_{22}y+H_{23}}{H_{31}x+H_{32}y+H_{33}}),$$ (1)

where D(x,y) are the new pixel coordinates of the false-color image, S(x,y) are the old coordinates, and H is the homography.

### 2.2 Light Sources

One commercially available light source: (a) halogen 3900 (Illumination Technologies, Liverpool, USA) as well as two customized ones: (b) Xenon 300 without near-infrared filter and (c) power LED 175 NIR [(b) and (c), KARL STORZ SE & Co. KG, Tuttlingen, Germany] were used for object illumination. The light source (c) emits in the visible (similar to Power LED 175) and near-infrared wavelength ranges. The intensity distribution of the three different light sources and white references were measured under the same conditions. At first, the exposure time and gain of the bwS were adjusted at centered entrance slit position and 5-cm object distance with the LED NIR light source at 80% of its maximum power. Exposure time and gain were constant for all measurements. Second, the power of each light source was increased until the measured intensities at 650 nm were equal for all three light sources. This resulted in an adjusted light source power of 40% for LED NIR, 6% for xenon, and 50% for halogen. During fixed motor position, the bwS provides 480 spectra in the spatial dimension. The mean intensity is calculated from the 100 centered spectra after subtraction of the black level. Moreover, the signal-to-noise ratio (SNR) of the laparoscopic system was estimated at a 5-cm object distance with the LED NIR (power: 80%) and xenon (power: 6%) light source. Similarly, the SNR of the HSI system for open surgery was estimated at a 50-cm object distance with a centered entrance slit position. The SNR was calculated according to

$$\mathrm{SNR}=20\times \log\;\frac{I_{\overset{¯}{1000}}}{\left|I-I_{\overset{¯}{1000}}\right|},$$ (2)

where $I_{\overset{¯}{1000}}$ is the average of 1000 acquired raw images and I is one single image. The resulting 480×100 (spatial×spectral) SNR array was averaged in the spatial dimension to yield the SNR over the wavelength for the sensor border (line 0 to 10), center (line 240 to 250), and all lines.

### 2.3 Reference Objects and Human Tissue Resectate

**Fig. 2:**
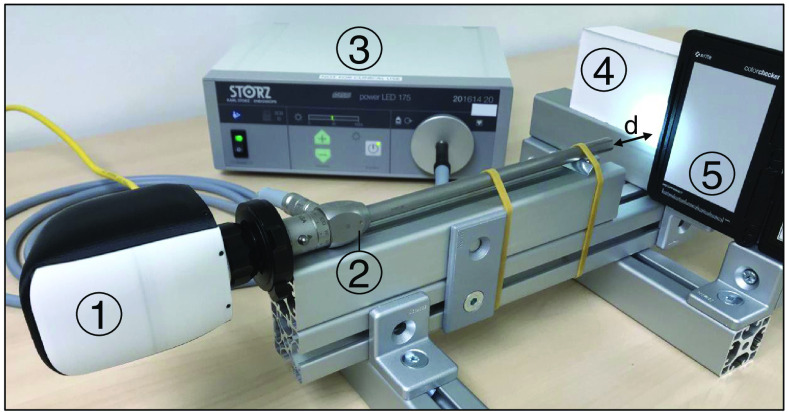
Setup for measurements with the (1) laparoscopic hyperspectral camera, (2) rigid laparoscope, (3) custom near-infrared LED light source, (4) white tissue phantom, and (5) ColorChecker. Working distance (d) is controlled manually by moving the mount of (4) and (5) along the guide rail.

For white and color references the ColorChecker (X-Rite, Grand Rapids, Michigan), white paper and a tissue phantom with scattering properties similar to human tissue were used. The latter consists of silicone and barium sulfate in a ratio of 30:7 without additional absorbers. Figure 2 shows the laboratory set up with the laparoscope, light source, and white references. Different realistic object distances were evaluated for the laparoscopic HSI system at 5- to 10-cm distances in 1-cm steps. The spatial resolution was evaluated with the 1951 United States Air Force (USAF) resolution test chart at 5- and 10-cm object distances by calculating the Michelson contrast of the resolution elements at 500 and 800 nm. A sine curve was fitted to the intensity profile and the maximum and minimum intensities for the determination of the Michelson contrast were obtained. The used Michelson contrast cut off was 20%. Furthermore, measurements were performed during abdomino-thoracic esophageal resection in a patient with Barrett’s Esophagus (Siewert classification: adenocarcinoma of esophagogastric junction type I-II, neoadjuvant chemotherapy). Therefore, the resectate was placed in the laparoscopic trainer Laparo Advance (Laparo, Wroclaw, Poland) and the laparoscope was attached to a holding arm during HSI. The ambient light was switched off during all measurements to avoid spectral artifacts.

### 2.4 Reference HSI System for Open Surgery

The data acquired with the laparoscopic HSI system were compared to the results obtained with a commercially available medical HSI system (TIVITA^®^ Tissue, Diaspective Vision GmbH, Am Salzhaff, Germany). This system was previously described by Kulcke et al.^17^ and is not suitable for endoscopic use. However, both HSI systems use pushbroom scanning and produce three-dimensional arrays with the same size, spectral resolution, and wavelength range. This allows the calculation of false-color images representing physiologic tissue parameters. Holmer et al.^18^ introduced the tissue hemoglobin index for skin that is multiplied by 0.5 to derive the organ hemoglobin index (OHI). OHI false-color images were calculated from the spectral data of a human tissue resectate to quantify the hemoglobin content from 0 to 100. The TIVITA camera was placed at a 50-cm distance to the object and six integrated halogen spots were used for illumination. For the evaluation of spectral comparability, reflectance spectra of a ColorChecker target were recorded with the TIVITA and the laparoscopic HSI system at 50-and 10-cm measurement distances, respectively. To get realistic results, the same camera settings as for the SNR measurements were used and were not adjusted for the larger object distance.

### 2.5 Data Preprocessing

The reflectance $I_{R}$ of the acquired spectral data was determined for every pixel (x,y) and wavelength (λ) according to

$$I_{R}=\frac{\left(I_{0}-I_{\text{dark}}\right)}{\left(I_{\text{white}}-I_{\text{dark}}\right)},$$ (3)

where $I_{\text{dark}}$ is the dark pattern without illumination, $I_{0}$ is the acquired intensity, and $I_{\text{white}}$ is the intensity measured on a white reference. $I_{\text{white}}$ was determined for every used object distance on white paper. The absorbance A was calculated by $A=-\log_{10}(I_{R})$.

For the calculation of OHI, the following equation was used:

$$\mathrm{OHI}=0.5\times \frac{\frac{{\overset{¯}{A}}_{530\ldots 590\;\mathrm{nm}}}{{\overset{¯}{A}}_{785\ldots 825\;\mathrm{nm}}}-a}{b-a},$$ (4)

where $\overset{¯}{A}$ is the average of the absorbance in the given wavelength range. The calibration values a and b are used to scale the OHI in the range from 0 to 100.

## 3 Results

**Fig. 3:**
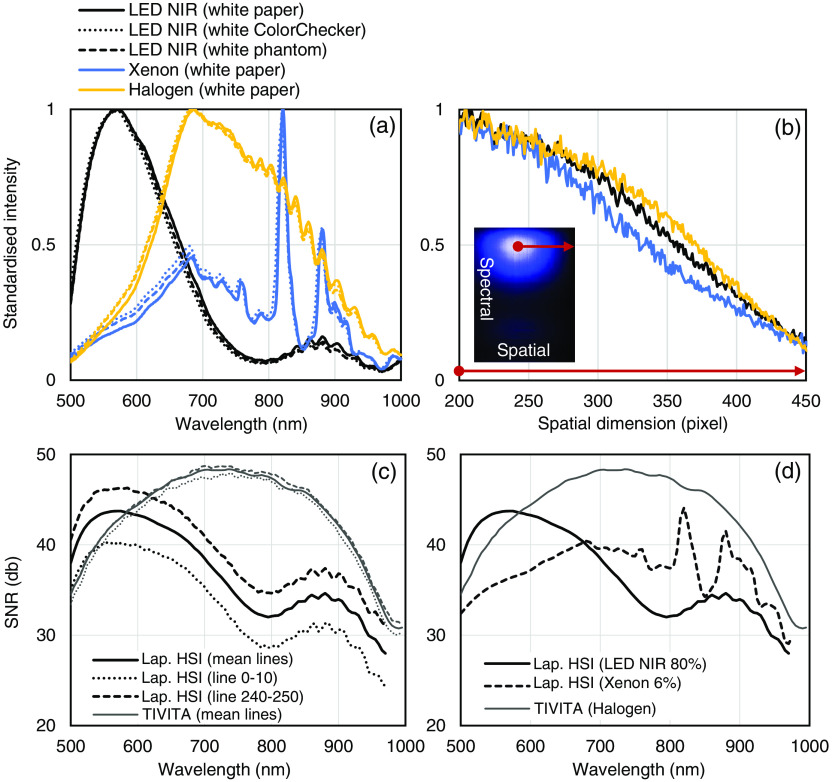
Spectral characteristic of the laparoscopic HSI system for different light sources (5-cm object distance): (a) standardized intensity of the LED NIR, xenon, and halogen light sources for three different white references (dotted lines represent the ColorChecker and dashed lines represent the white phantom). (b) Intensity decrease from illumination spot center to the border of the image for LED NIR (575 nm), xenon and halogen (685 nm). (c) SNR of the laparoscopic HSI system with LED NIR light source compared to the TIVITA system. The mean SNR of sensor lines 0 to 10, 240 to 250, and all lines in the spatial dimension are plotted for both systems. (d) Mean SNR of all sensor lines for the laparoscopic HSI (LED NIR and xenon light source) compared to the TIVITA system (halogen light).

Standardized intensities of the three different light sources and white references are shown in Fig. 3(a). Ripples in the measured intensities above 800 nm are due to the spectral efficiency of the sensor and corrected during white balancing. The spatial intensity decrease of the light sources was evaluated during the same measurement from the illumination spot center to the border of the image. An intensity drop about 50% at 150 pixels distance from the spot center was observed for the LED NIR (575 nm), xenon and halogen (685 nm) light source [Fig. 3(b)]. The mean SNR of all sensor lines was 30 to 43 dB in the spectral range from 500 to 950 nm as illustrated in Figs. 3(c) and 3(d). Due to the intensity decrease at the image borders, up to 7 dB difference in the spatial dimension was observed. The TIVITA system with halogen lighting unit (50-cm object distance) showed an overall higher SNR compared to the laparoscopic system with LED or xenon light source. However, in the spectral range with high absorption coefficients of hemoglobin (<600  nm), the LED NIR light source provided a higher SNR.

**Fig. 4:**
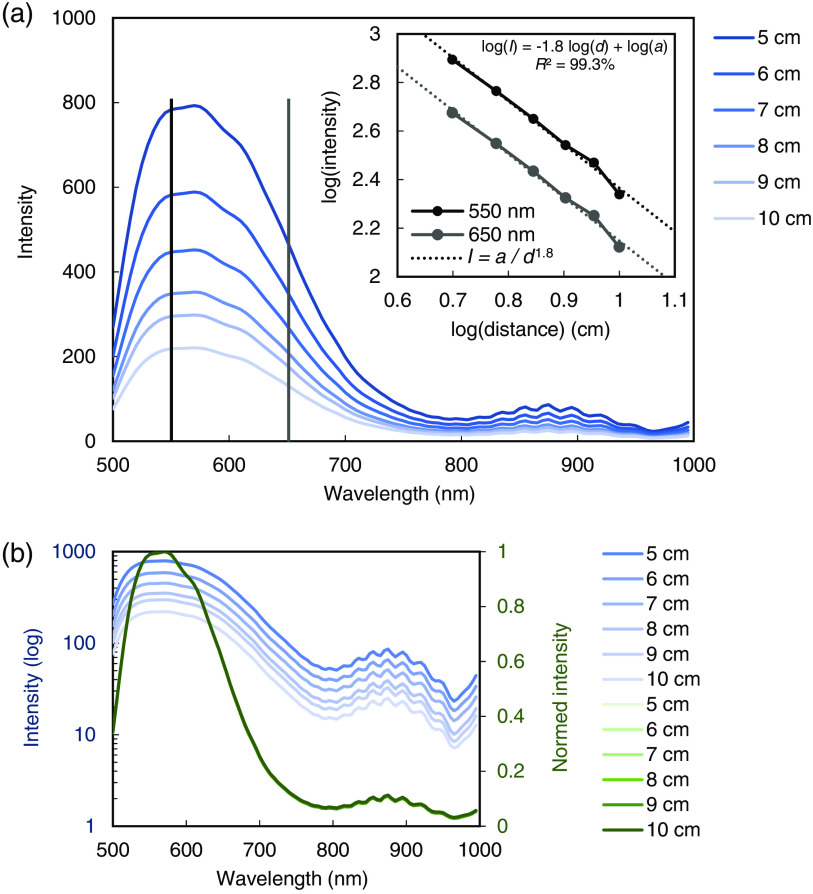
Intensity decrease in the image center measured with the laparoscopic HSI system and LED NIR light source at different object distances: (a) the measured intensities are approximately following the inverse-square law ($\text{intensity}\propto 1/{\text{distance}}^{2}$). (b) Logarithmic scale and normalized intensities indicate that the shape of the spectra is distance-independent.

The spectral distribution of the LED NIR light source measured at 5- to 10-cm distances in 1-cm steps is shown in Fig. 4. An intensity decrease proportional to ${1/\text{distance}}^{1.8}$ was observed, which approximately follows the inverse-square law for point sources.

At a 5-cm object distance and 500 nm, the spatial resolution was 320  μm in the x dimension and 160  μm in the y dimension. The spatial resolution approximately halved (630  μm in the x dimension and 315  μm in the y dimension) for a doubled object distance (10 cm). A decreased spatial resolution was observed in the near-infrared range (800 nm) for the y dimension (500  μm) though it remained constant for the x dimension (scanning direction).

**Fig. 5:**
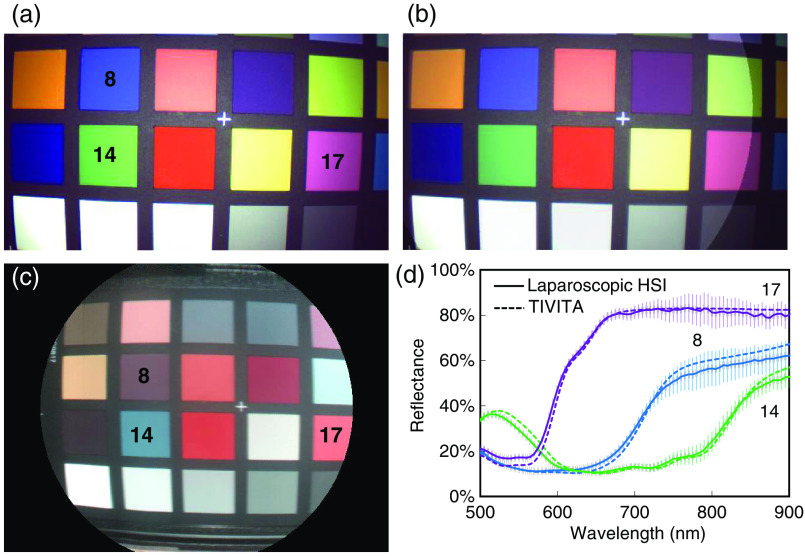
Simultaneous video and HSI of the ColorChecker target: (a) video from the color image sensor (rgbS). (b) Registration and semitransparent overlay of the rgbS video and color image from HSI after one-time calculation of the homography. (c) Color image calculated during hyperspectral data acquisition. Pseudocolors are calculated from the measured reflectance between 530 and 725 nm, due to the lower SNR of the TIVITA system below this range. (d) Reflectance of three exemplary ColorChecker patches measured with the laparoscopic HSI and TIVITA system. Error bars indicating the standard deviations for the patches recorded with the laparoscopic system. Standard deviations for the TIVITA system are <2% and omitted for clarity.

In Fig. 5, a static image of the color video from the color image sensor (rgbS), a color image calculated from HSI data of the bwS, and the registration of both images with semitransparent overlay are shown. The average root-mean-square error (RMSE) of the reflectance (500 to 900 nm) from 11 ColorChecker patches measured with the laparoscopic and TIVITA system was 0.03 (±0.02). Three exemplary reflectance curves of these patches are given in Fig. 5(d) for both systems.

**Fig. 6:**
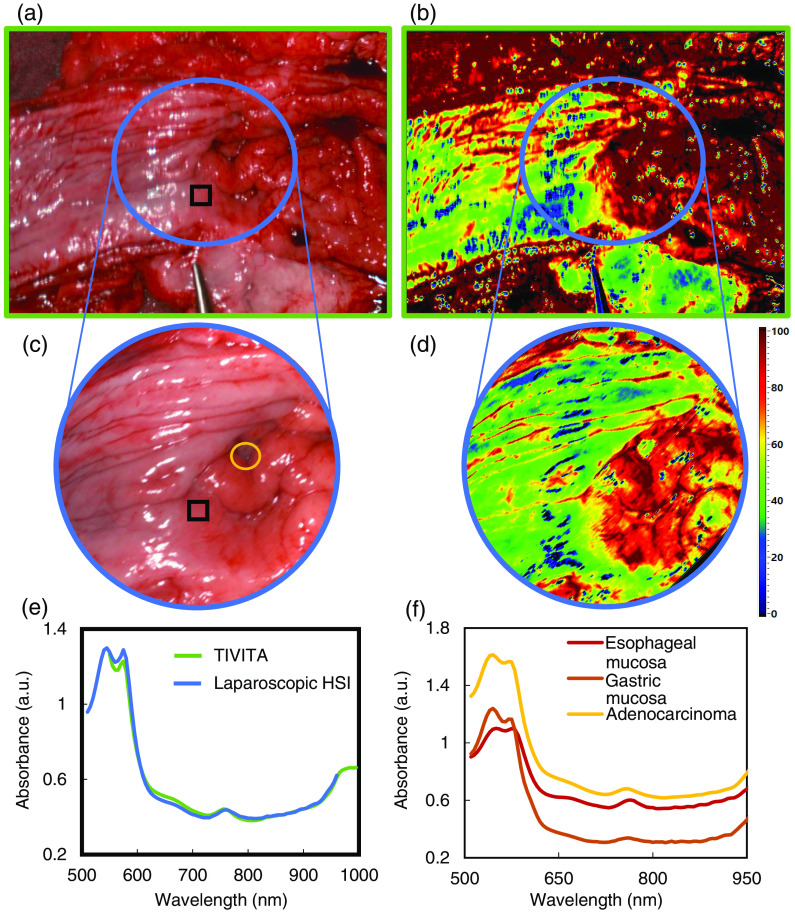
Intraluminal HSI of the resected adenocarcinoma of the esophagogastric junction: color image and OHI calculated from spectral data acquired with (a), (b) TIVITA and (c), (d) laparoscopic HSI system. The corresponding FOV is labeled as a blue circle. (e) Mean absorbance spectrum of the pixels inside the black square for both HSI systems. (f) Mean absorbance spectrum of adenocarcinoma (pixels inside yellow circle), esophageal and gastric mucosa (outside of the FOV) measured with the laparoscopic HSI system and labeled by a surgeon according to visual and haptic perception.

The laparoscopic HSI system was successfully used in clinical set up for measurements of a resected adenocarcinoma of the esophagogastric junction from a patient with Barrett’s Esophagus. Data were acquired from extra- and intraluminal immediately after abdomino-thoracic esophageal resection. The corresponding structures in the color images acquired with the TIVITA and laparoscopic HSI system were matched postoperatively. Figure 6 shows the color images and OHI false-color images from the TIVITA and the laparoscopic HSI system. In addition, the mean absorbance spectra inside a corresponding ROI are illustrated. The data are plotted from 500 to 960 nm for the laparoscopic system and 550 to 1000 nm for TIVITA because of the low intensities outside this range. The RMSE of the two mean absorbance spectra inside the region of interest (ROI) is 0.02 (550 to 960 nm). For eight randomly selected ROIs, the average RMSE is 0.09 (±0.05).

## 4 Discussion

The intraoperative estimation of tissue perfusion and differentiation is essential during most laparoscopic procedures. HSI can support visual decision making, but is not yet ready for routine laparoscopic use. An HSI system with simultaneous high-resolution video for minimal-invasive surgery may reduce the risk of perfusion related complications and assist the identification of lesions and risk structures.

In this work, a laparoscopic system capable of spectral imaging from 500 to 1000 nm with high spatial and spectral resolution in 4.6 s was described. The raw spectral data are processed and visualized in real time during acquisition, hence no additional time is needed. Furthermore, the handheld device provides high-resolution video during HSI and allows pushbroom imaging without the previously reported limitations of moving the object or the laparoscope. The registration of false-color images derived from the hyperspectral data (e.g., perfusion maps) and high-resolution video provides augmented visual information. The measured ColorChecker reflectance data were consistent with a commercially available medical HSI device for the practical object distance of 10 cm. Moreover, corresponding regions of the resected esophagogastric junction showed the same spectral signature for both systems although the light source and measurement distance were different.

Additionally, OHI false-color images of the resected tissue from the open and laparoscopic HSI system showed the same qualitative results. Quantitative deviations of OHI were observed and could be due to the time delay between measurements, repositioning in the laparoscopic trainer, and glare artifacts, which needs to be investigated more closely.

Only minor differences were observed for the spectral shape of the three different white references, however, variations in absolute intensity were present and must be considered when comparing reflectance values. Even though the used light sources slightly differed in their spatial distribution, all showed a strong intensity decline in the FOV. A homogenous illumination would improve the SNR at the image borders.

The entrance slit can be moved automatically by the integrated motor to scan the image or can be stopped at any position if desired. This design also allows manual scanning, such as that recently described by Yoon et al.^16^ for flexible endoscopy.

Currently, the SNR in the near-infrared range is limited by the available light sources and the transmission of the used laparoscopes. Work is in progress to solve this problem. The size of the system is already comparable to usual laparoscopic cameras. However, the housing could become even smaller by removing parts of the processing hardware. To avoid motion artifacts, the laparoscope was attached to a holding arm during scanning. Movements of the tissue, for instance due to breathing, cannot be prevented and thus should be aimed at further reduction of HSI acquisition time.

In the authors’ opinion, rigid laparoscopic HSI has great potential for fast and repeatable quantification of tissue perfusion as reported by Clancy et al.^21^ Although the measured reflectance data agreed with the results from the TIVITA system, studies on perfused tissue need to verify that the approved perfusion indices are valid for laparoscopic use.

Laparoscopic HSI in the visible and near-infrared range with short processing time and simultaneous video is feasible and fulfills the requirements for routine clinical use. Studies with more clinical data are needed to evaluate the benefits of laparoscopic HSI for intraoperative applications.

## 5 Conclusions

An HSI system with high spatial and spectral resolution in the visible and near-infrared range and simultaneous color video for minimally invasive surgery was presented. Reference objects and resected tissue were used to show the consistency of the measured data from the laparoscopic HSI and the approved TIVITA system. The compact handheld design, rapid HSI, and high-resolution video at the same time enable high clinical practicability. This work may support the translation of HSI to routine laparoscopic surgery. Future studies on the added value of laparoscopic HSI for the patient outcome are needed.
